# Supplementary material for: Risk of major autoimmune diseases in female breast cancer patients: A nationwide, population-based cohort study
Source: PLoS One. 2019 Sep 19;14(9):e0222860. doi: 10.1371/journal.pone.0222860 (PMC6752851; doi:10.1371/journal.pone.0222860)
Supplement: Attachment — Submitted filename: Review on AID and BC in Taiwan.docx [file pone.0222860.s001.docx]

Review on «Risk of Major Autoimmune Diseases in Patients with Female Breast Cancer: a nationwide, Population-based Cohort Study”.

**General comments**

The idea is novel, and inspired by very up-to-date science: the importance of immune responses in breast cancer. In the US, there about 300 clinical studies registered on this issue. This study illustrates the potential for immunotherapy.

The paper needs English editing to give an impression of serious science. Too many typing errors that I can correct them all. I also think many sentences can be written better, for example, I think this heading is better “Risk of Major Autoimmune Diseases in female breast Cancer: a Population-based Cohort Study”. First sentence in the abstract “Breast cancer is one of common malignancy among women” illustrates the low level of proficiency in English.

Method: The method needs to be better described and justified. Why not using all the data in NHRID? Then they need not to match and later adjust for covariates. This is a case-control cohort study. Breast cancers are recorded during 2007-13, while non-breast cancer patients are recorded during the period 2003-2013. This can make problems, I think. If not, please explain. Why not use all the population in NHRID, split them in breast cancer patients and not breast cancer patients at start, and then follow them up until disease (and censor them). Then you have a prospective cohort study, and less variables to adjust for. The potential risk of biased outcome/false positive reporting in this study is related to the adjustment.

In Table 2, I would like to see estimates before adjustment of adjuvant therapy. Adjustment for CIC and adjuvant therapy may generate over-adjustment. You adjust for the same disease twice, unless you think that adjuvant therapy directly influence on the risk of autoimmune disease.

**Minor comments**

Abstract, Method: the ratio is 1:4, I think. You are not matching on sex, I think. You are excluding males.

Page numbers are missing. Would be fine with line numbers too.

Introduction, second sentence. Delete “prevalence”. Only focus on incidence, because women live longer than men do. Thus, comparing prevalence rates is not meaningful.

Last sentence, last paragraph, introduction (especially 6^th^ last line): bad English, too long sentence. Furthermore, the aim/hypothesis to study has already been presented in the previous paragraph.

Data source, first paragraph, forth sentence: The word “claims” is not necessary. Sufficient to write “… comprehensive data regarding…” Last sentence: “provides data” and not “databases” to researchers.

I think “catastrophic illness” is not the proper word to use. “Possible fatal illness”, is probably better.

Definition of female breast cancer patients: CIC is not defined. And not MAD either, in the next paragraph.

Potential confounders: gender??? I suggest excluding all male breast cancer patients from start and only study women. Then adjusting for gender is not necessary.

Statistical analyses: I think you have done a case-control cohort study.
