# Supplementary material for: Risk of major autoimmune diseases in female breast cancer patients: A nationwide, population-based cohort study
Source: PLoS One. 2019 Sep 19;14(9):e0222860. doi: 10.1371/journal.pone.0222860 (PMC6752851; doi:10.1371/journal.pone.0222860)
Supplement: Attachment — Submitted filename: Response to Reviewers.docx [file pone.0222860.s002.docx]

Response to reviewers:

Dear Editors-in-Chief and Reviewers,

Reviewer #1: This study makes use of the large Taiwanese National Health Insurance Research Database (NHIRD) to compare the incidence rates of autoimmune diseases in breast patients and propensity score matched non-breast cancer individuals. The study design is well powered, data analysis is clean, and results are unambiguous.

I only have some minor comments—
1) In title the expression “in patients with female breast cancer” is awkward. Please consider rephrase it, for example, as “in female patients with breast cancer”?

Response: We had rephrased it as ‘in female breast cancer’.

2) There is a typo in Abstract “1:4” instead of “1:40”.
Response: We correct it.

Reviewer #2: General comments

**General comments**

The idea is novel, and inspired by very up-to-date science: the importance of immune responses in breast cancer. In the US, there about 300 clinical studies registered on this issue. This study illustrates the potential for immunotherapy.

The paper needs English editing to give an impression of serious science. Too many typing errors that I can correct them all. I also think many sentences can be written better, for example, I think this heading is better “Risk of Major Autoimmune Diseases in female breast Cancer: a Population-based Cohort Study”. First sentence in the abstract “Breast cancer is one of common malignancy among women” illustrates the low level of proficiency in English.

Response: We have rephrased the title. Changes have been made pertaining to language and grammar.

Method: The method needs to be better described and justified. Why not using all the data in NHRID? Then they need not to match and later adjust for covariates. This is a case-control cohort study. Breast cancers are recorded during 2007-13, while non-breast cancer patients are recorded during the period 2003-2013. This can make problems, I think. If not, please explain. Why not use all the population in NHRID, split them in breast cancer patients and not breast cancer patients at start, and then follow them up until disease (and censor them). Then you have a prospective cohort study, and less variables to adjust for. The potential risk of biased outcome/false positive reporting in this study is related to the adjustment.

Response:

1. Because we were unable to get all the data in NHIRD, the only way to get matched non-breast cancer individuals was to use the one million representative population in the LHID2000. We have added a statement in Line 8 of page 6: “Because we were unable to get all the data in NHIRD, we selected the non-breast cancer comparison cohort from the population in the LHID2000.”
2. The periods of datasets for the female breast cancer cohort and the comparison cohort are the same (i.e., from 2003 to 2013). To identify new cases of breast cancer during 2007–2013, we excluded patients who had a diagnosis of breast cancer before 2007. To eliminate the differences of diagnosis and treatment in different era, the comparison cohort was also matched for the diagnosis date of female breast cancer patients.

In Table 2, I would like to see estimates before adjustment of adjuvant therapy. Adjustment for CIC and adjuvant therapy may generate over-adjustment. You adjust for the same disease twice, unless you think that adjuvant therapy directly influence on the risk of autoimmune disease.

Response:

1. Chemotherapy and hormone therapy may affect lymphocytes and other immune cells, affecting the risk of autoimmune disease.
2. CCI was used to represent the level of general comorbid medical conditions. For female breast cancer patients, breast cancer was not included for calculating CCI. Therefore, we did not adjust for the same disease twice. We ahve added a statment in Line 8 of page 9: “For female breast cancer patients, breast cancer was not included for calculating CCI. ”

**Minor comments**

Abstract, Method: the ratio is 1:4, I think. You are not matching on sex, I think. You are excluding males.

Response: Since we have excluded males, we did not match the sex. We have removed “sex” in the list of matched variables.

Page numbers are missing. Would be fine with line numbers too.

Response: We have added continuous page and line numbers.

Introduction, second sentence. Delete “prevalence”. Only focus on incidence, because women live longer than men do. Thus, comparing prevalence rates is not meaningful.

Response: We have corrected it according to your suggestion.

Last sentence, last paragraph, introduction (especially 6^th^ last line): bad English, too long sentence. Furthermore, the aim/hypothesis to study has already been presented in the previous paragraph.

Response: We have revised the statement for concision a follows. “The aim of this study was to investigate the risks of major autoimmune diseases in female breast cancer patients using the NHIRD.” We have deleted a redundant statement regarding the hypothesis.

Data source, first paragraph, forth sentence: The word “claims” is not necessary. Sufficient to write “… comprehensive data regarding…” Last sentence: “provides data” and not “databases” to researchers.

Response: We have corrected it based on your suggestions.

I think “catastrophic illness” is not the proper word to use. “Possible fatal illness”, is probably better.

Response: We have used “possible fatal illness” to describe the characteristics of these diseases. However, the official name of the certificate for patients with these “possible fatal illness” is “catastrophic illness certificate” in Taiwan.

Definition of female breast cancer patients: CIC is not defined. And not MAD either, in the next paragraph.

Response:

1. To avoid confusion with CCI, we have removed the abbreviation of CIC and used “catastrophic illness certificate” in the manuscript.
2. Because MAD is not a common abbreviation, we have deleted this abbreviation and used the entire definition “major autoimmune disease” in manuscript.

Potential confounders: gender??? I suggest excluding all male breast cancer patients from start and only study women. Then adjusting for gender is not necessary.

Response: Because we have excluded males, we have removed “gender” in the list of potential confounders.

Statistical analyses: I think you have done a case-control cohort study.

Response: This study is a retrospective cohort study and not case-control study. Therefore, we have used the Cox regression analysis.
