# Supplementary material for: Risk of major autoimmune diseases in female breast cancer patients: A nationwide, population-based cohort study
Source: PLoS One. 2019 Sep 19;14(9):e0222860. doi: 10.1371/journal.pone.0222860 (PMC6752851; doi:10.1371/journal.pone.0222860)
Supplement: Attachment — Submitted filename: Review on PONE-R1-2019 .docx [file pone.0222860.s003.docx]

**Review on PONE-D-19-1596 (Risk of major Autoimmune Diseases in Female Breast Cancer Patients: A Nationwide, Population-based Cohort Study).**

The paper has improved a lot. I am Ok with the statistics (I have PhD in mathematical statistics). I have numerous suggestions on how to make the paper easier to read, including editing the English language (English is not my native language).

Abstract, lines 12-14: ….selected females diagnosed with breast cancer in the period 2007 to 2013 into a control group.

Abstract, line 15. Delete sex. Not necessary to match on sex when you only study women.

Abstract, lines 19-21. ….For SLE, the incidence rates were 2.3 (BC group) vs 10.0 (control group) per 100,000 women year; for RA rates were 19.3 (BC group) vs 42.7 (control group) per 100,000 women year; and for SS rates were 20.5 (BC group) vs 38.2 (control group) per 100,000 women year.

Do not use 10 raised to the power of fifth in a medical journal.

Abstract, page 3 , lines 1-4: After adjusting for potential confounders, the hazard ratio (HZ) with corresponding confidence interval (CI) for females breast cancer patients vs control group were 0.04 (95% CI: 0.01-0.24) for SLE; 0.03 (95% CI: 0.02-0.04) for RA; and 0.21 (95% CI: 0.09-0.48) for SS.

Page 4, line 2: ….one of the most common….

Page 5, lines 13-14: The data set were extracted from the NHIRD database using data from the period 2003 to 2013.

Page 6, lines 5 -6: …..,including data on both inpatients and outpatients,….

Page 6, lines 9-10:….,we selected the non-breast cancer control group from the LHID cohort.

Page 7, line 13: delete leaving and write “emigration”. Is there any other way of leaving the database than emigration or death?

Page 8, line 6: …,as the control group.

Page 8, line 10: including 409,340 female non-breast cancer individuals before matching.

Page 9, line 17…,by using Student’s t-test,….by using Pearson’s…

Page 9, line18; confidence interval has been defined above.

Page 10, line 2: HZ has been defined before.

Page 10, lines 13-14: …. ,the incidence rate of SLE was 2.3 per 100,000 women years, the incidence rate of RA was 19.3 per 100,000 women years, the incidence rate of SS was 20.1 per 100,000 women years and the incidence rate of DMtis/PM was 2.3 per 100,000 women years.

Page 10, lines 15-16: Do as above.

Page 10, line 19: , the hazard ratio (HZ) with corresponding confidence interval (CI) for females breast cancer patients vs control group were 0.04 (95% CI: 0.01-0.24) for SLE; 0.03 (95% CI: 0.02-0.04) for RA; and 0.21 (95% CI: 0.09-0.48) for SS.

Page 12, line 5: …had a significant lower risk….

Page 13, line 6. Do not use the word “claim data”. This is register data.

Page 15, Table 1. Spelling error in the line under “Concomitant medication”. FiRst

Page 15, Table 1: “Time to first SLE”… The number look like real numbers with percentage in parenthesis and not life times. Please correct or explain.
