# Supplementary material for: Risk of major autoimmune diseases in female breast cancer patients: A nationwide, population-based cohort study
Source: PLoS One. 2019 Sep 19;14(9):e0222860. doi: 10.1371/journal.pone.0222860 (PMC6752851; doi:10.1371/journal.pone.0222860)
Supplement: Attachment — Submitted filename: Response to Reviewers.docx [file pone.0222860.s004.docx]

Response to reviewers:

Dear Editors-in-Chief and Reviewers,

Reviewer #2:

Abstract, lines 12-14: ….selected females diagnosed with breast cancer in the period 2007 to 2013 into a control group.

Response: We rephrased abstract. We identified newly-diagnosed female breast cancer patients and randomly selected females without breast cancer in the period 2007 to 2013 into a control group.

Abstract, line 15. Delete sex. Not necessary to match on sex when you only study women.

Response: We have deleted ‘sex’.

Abstract, lines 19-21. ….For SLE, the incidence rates were 2.3 (BC group) vs 10.0 (control group) per 100,000 women year; for RA rates were 19.3 (BC group) vs 42.7 (control group) per 100,000 women year; and for SS rates were 20.5 (BC group) vs 38.2 (control group) per 100,000 women year. Do not use 10 raised to the power of fifth in a medical journal.

Response: We rephrased abstract.

Abstract, page 3 , lines 1-4: After adjusting for potential confounders, the hazard ratio (HZ) with corresponding confidence interval (CI) for females breast cancer patients vs control group were 0.04 (95% CI: 0.01-0.24) for SLE; 0.03 (95% CI: 0.02-0.04) for RA; and 0.21 (95% CI: 0.09-0.48) for SS.

Response: We rephrased abstract: After adjusting for potential confounders, the hazard ratios (95% confidence intervals) for female breast cancer patients vs. control group were 0.04 (0.01–0.24) for SLE; 0.03 (0.02–0.04) for RA; and 0.21 (0.09–0.48) for SS.

Page 4, line 2: ….one of the most common….

Response: We rephrased introduction: Breast cancer is one of the most common malignancies among women.

Page 5, lines 13-14: The data set were extracted from the NHIRD database using data from the period 2003 to 2013.

Response: We rephrased method.

Page 6, lines 5 -6: …..,including data on both inpatients and outpatients,….

Response: We rephrased method.

Page 6, lines 9-10:….,we selected the non-breast cancer control group from the LHID cohort.

Response: We rephrased method.

Page 7, line 13: delete leaving and write “emigration”. Is there any other way of leaving the database than emigration or death?

Response: We rephrased method. We defined the censor date as December 31, 2013 (the last date of the database used) or the date of withdrawal from the NHI for emigration or death.

Page 8, line 6: …,as the control group.

Response: We rephrased method.

Page 8, line 10: including 409,340 female non-breast cancer individuals before matching.

Response: We rephrased method.

Page 9, line 17…,by using Student’s t-test,….by using Pearson’s…

Response: We rephrased method.

Page 9, line18; confidence interval has been defined above.

Response: Because CI was not defined before in the revised manuscript, so we kept the definition of HZ here.

Page 10, line 2: HZ has been defined before.

Response: Because HZ was not defined before in the revised manuscript, so we kept the definition of HZ here.

Page 10, lines 13-14: …. ,the incidence rate of SLE was 2.3 per 100,000 women years, the incidence rate of RA was 19.3 per 100,000 women years, the incidence rate of SS was 20.1 per 100,000 women years and the incidence rate of DMtis/PM was 2.3 per 100,000 women years.

Response: We rephrased results.

Page 10, lines 15-16: Do as above.

Response: We rephrased results.

Page 10, line 19: , the hazard ratio (HZ) with corresponding confidence interval (CI) for females breast cancer patients vs control group were 0.04 (95% CI: 0.01-0.24) for SLE; 0.03 (95% CI: 0.02-0.04) for RA; and 0.21 (95% CI: 0.09-0.48) for SS.

Response: We rephrased results.

Page 12, line 5: …had a significant lower risk….

Response: We rephrased discussion.

Page 13, line 6. Do not use the word “claim data”. This is register data.

Response: We rephrased discussion.

Page 15, Table 1. Spelling error in the line under “Concomitant medication”. FiRst

Response: We correct spelling error.

Page 15, Table 1: “Time to first SLE”… The number look like real numbers with percentage in parenthesis and not life times. Please correct or explain.

Response: We correct Table 1.

Thank you for your helpful suggestion.
